# Determinants of quality of life in adults with epilepsy: a multicenter, cross-sectional study from Germany

**DOI:** 10.1186/s42466-023-00265-5

**Published:** 2023-08-03

**Authors:** Kai Siebenbrodt, Laurent M. Willems, Felix von Podewils, Peter Michael Mross, Michael Strüber, Lisa Langenbruch, Laura Bierhansl, Iris Gorny, Juliane Schulz, Bernadette Gaida, Nadine Conradi, Annika Süß, Felix Rosenow, Adam Strzelczyk

**Affiliations:** 1 Epilepsy Center Frankfurt Rhine-Main, Department of Neurology, Goethe-University and University Hospital Frankfurt, Frankfurt am Main, Germany; 2 LOEWE Center for Personalized Translational Epilepsy Research (CEPTeR), Goethe-University Frankfurt, Frankfurt am Main, Germany; 3 Department of Neurology, Epilepsy Center, University Hospital Greifswald, Greifswald, Germany; 4 Epilepsy Center Hessen and Department of Neurology, Philipps-University Marburg, Marburg, Germany; 5 Institute for Artificial Intelligence in Medicine, Philipps-University Marburg, Marburg, Germany; 6 Epilepsy Center Münster-Osnabrück, Department of Neurology with Institute of Translational Neurology, Westfälische Wilhelms-University, Münster, Germany

**Keywords:** Epilepsy, Depression, Quality of life, Seizure, QOLIE-31, Anti-seizure medication

## Abstract

**Background:**

Assessment of quality of life (QoL) has become an important indicator for chronic neurological diseases. While these conditions often limit personal independence and autonomy, they are also associated with treatment-related problems and reduced life expectancy. Epilepsy has a tremendous impact on the QoL of patients and their families, which is often underestimated by practitioners. The aim of this work was to identify relevant factors affecting QoL in adults with epilepsy.

**Methods:**

This cross-sectional, multicenter study was conducted at four specialized epilepsy centers in Germany. Patients diagnosed with epilepsy completed a standardized questionnaire focusing on QoL and aspects of healthcare in epilepsy. Univariate regression analyses and pairwise comparisons were performed to identify variables of decreased QoL represented by the overall Quality of Life in Epilepsy Inventory (QOLIE-31) score. The variables were then considered in a multivariate regression analysis after multicollinearity analysis.

**Results:**

Complete datasets for the QOLIE-31 were available for 476 patients (279 [58.6%] female, 197 [41.4%] male, mean age 40.3 years [range 18–83 years]). Multivariate regression analysis revealed significant associations between low QoL and a high score on the Liverpool Adverse Events Profile (LAEP; beta=-0.28, p < 0.001), Hospital Anxiety and Depression Scale – depression subscale (HADS-D; beta=-0.27, p < 0.001), Neurological Disorders Depression Inventory in Epilepsy (NDDI-E; beta=-0.19, p < 0.001), revised Epilepsy Stigma Scale (beta=-0.09, p = 0.027), or Seizure Worry Scale (beta=-0.18, p < 0.001) and high seizure frequency (beta = 0.14, p < 0.001).

**Conclusion:**

Epilepsy patients had reduced QoL, with a variety of associated factors. In addition to disease severity, as measured by seizure frequency, the patient’s tolerability of anti-seizure medications and the presence of depression, stigma, and worry about new seizures were strongly associated with poor QoL. Diagnosed comorbid depression was underrepresented in the cohort; therefore, therapeutic decisions should always consider individual psychobehavioral and disease-specific aspects. Signs of drug-related adverse events, depression, fear, or stigmatization should be actively sought to ensure that patients receive personalized and optimized treatment.

**Trial Registration:**

German Clinical Trials Register (DRKS00022024; Universal Trial Number: U1111-1252-5331).

## Background

Analysis of quality of life (QoL) has become a relevant indicator in many chronic neurological disorders due to their association with a reduction in personal independence and autonomy, treatment-related problems, and a reduced life expectancy [1]. QoL is a complex, multidimensional construct that describes the general well-being of an individual by outlining individual negative and positive aspects of life rather than just physical health [2]. Epilepsy is one of the most common chronic neurological disorders; it affects about 60 million people worldwide. Patients suffering from epilepsy experience spontaneous recurrent epileptic seizures, which manifest as a variety of symptoms that may affect all sensory modalities, consciousness, psychological state, and the motor system [3]. The diagnosis of epilepsy has an enormous impact on the QoL of patients, their relatives and caregivers [4, 5]. Epilepsy affects several dimensions of QoL, including physical, cognitive, psychobehavioral, and social aspects. Even though two-thirds of patients with epilepsy become seizure-free with anti-seizure medications (ASMs), they often suffer from numerous adverse events. About one-third of patients remain resistant to ASMs, which may lead to treatment discontinuation and further deterioration of QoL [6, 7]. Patients with epilepsy often need to be hospitalized due to the illness or seizure-related injuries [8]. In addition to epileptic seizures, many patients report chronically impaired cognitive function and psychiatric symptoms, such as mood disorders [9], and these typical symptoms are difficult to treat. Adults with epilepsy often suffer from additional stigma and discrimination that negatively affect their QoL [10, 11].

Physicians often underestimate the consequences of the diagnosis and treatment of epilepsy on the QoL of patients [12]. Therefore, the assessment and measurement of QoL have become a major subject of interest in clinical practice and research; measurement should be acceptable to patients, psychometrically driven, and administratively practical [13]. There are several methods of measuring QoL in epilepsy patients; two of the most common scales are the generic European Quality of Life 5 Dimensions inventory (EQ-5D) and the 31-item health-related Quality of Life in Epilepsy inventory (QOLIE-31). Despite its length, the QOLIE-31 more accurately represents QoL than shorter assessments, such as the EQ-5D and the QOLIE-10, especially in severely affected patients [14], while the EQ-5D has the advantage of using normative data, which is available for the general population [15].

This study aimed to detect the most relevant factors that are negatively associated with QoL in adults with epilepsy, considering the recent approval of many new ASMs and the limited QoL data available for adults with epilepsy and to better understand certain aspects of epilepsy treatment to provide patients with individualized epilepsy therapy.

## Methods

### Study settings, patients, and design

This multicenter, cross-sectional Epi2020 study was conducted at four different epilepsy centers in Germany (Frankfurt am Main, Marburg, Münster, and Greifswald) that offer specialized in- and outpatient care for patients with epilepsy, epileptic encephalopathies, and syndromes associated with epilepsy [16, 17]. Patients were instructed to complete a standardized questionnaire focusing on their QoL and other healthcare-related aspects of epilepsy. All adult patients with a confirmed diagnosis of epilepsy were eligible for participation between 10/2020 and 12/2020. Written informed consent was mandatory and was provided before enrolment. The study was registered with the German Clinical Trials Register (DRKS00022024; Universal Trial Number: U1111-1252-5331) and approved by the ethics committee of the Goethe-University Frankfurt (reference number 19–440). The Strengthening the Reporting of Observational Studies in Epidemiology (STROBE) and Reporting of studies Conducted using Observational Routinely collected health Data (RECORD) guidelines were closely followed [18, 19].

### Scores and metrics

To objectivize health-related QoL, the questionnaire included the QOLIE-31 inventory, which is well-established in this field and represents the gold standard for QoL assessment in epilepsy patients [20, 21]. It consists of 30 questions and one visual analog scale addressing seven subcategories (worry about seizures, overall QoL, emotional well-being, energy and fatigue, cognitive impairment, medication effects, and social function). Analysis of the QOLIE-31 was performed according to the recommendations of the QOLIE development group to determine the overall score and T-score [22]. In addition, the generic EQ-5D was evaluated to compare the results of people with epilepsy (PWE) to normative data from the general population [23].

Therapy-related adverse events were measured using the Liverpool Adverse Events Profile (LAEP), which features a list of 19 symptoms that are rated using a Likert scale. The LAEP is an established tool that has been used in several research settings [24, 25]. The questionnaire also included the Neurological Disorders Depression Inventory for Epilepsy (NDDI-E), which is a brief and reliable depression screening instrument for epilepsy patients; a cutoff score of ≥ 14 indicated the probable presence of depression [26]. In addition, the Hospital Anxiety and Depression Scale (HADS) was assessed, which represents a well-established self-assessment procedure for screening anxiety and depressive symptoms with high utility in clinical practice. The total score can be used, or it can be divided into individual subscales for depression (HADS-D) and anxiety (HADS-A). Using the subscales, a score of ≥ 8 points was considered an indicator of depression or an anxiety disorder [27]. The revised Epilepsy Stigma Scale (rESS) was used to assess the extent of stigmatization. It consists of three questions that are answered using a Likert scale from 0 to 3 for each question, resulting in a total score of 0–9, with 7–9 indicating high stigma, 1–6 indicating mild-to-moderate stigma, and 0 indicating no stigma [28]. The Seizure Worry Scale (SWS) is a two-item instrument that uses a Likert scale from 0 to 3. The total score ranges from 0 to 6; 3–6 reflects moderate-to-high seizure worry and 0–2 represents no-to-mild seizure worry [29].

To correlate the measured items with sociodemographic- and disease-related aspects, several factors were assessed. Participants reported their sex, age, body mass index (BMI), seizure frequency, type and dose of ASM, concomitant diseases and medications, relationship status, occupational status, and whether they had children and a certificate of disability. The treating physician provided additional information about the type of epilepsy and the epilepsy duration.

### Statistical analysis

Data input, statistical workup, and graphical representation were performed using SPSS (version 27 or higher, IBM Corporation, Armonk, NY, US) and GraphPad Prism 9 (GraphPad Software Inc., La Jolla, CA, US). A p-value below 0.05 was considered statistically significant.

Descriptive analyses were conducted for clinical and sociodemographic characteristics and the evaluated scores. To identify variables of decreased QoL, the association between clinical and sociodemographic variables and the QOLIE-31 scores were assessed using univariate linear regression analysis in case of numerical data, in non-numerical data the Kruskal–Wallis test with Bonferroni correction was employed. Pairwise comparisons were performed using the unpaired t-test.

To evaluate potential predictors of QoL (dependent variable: QOLIE-31 overall score), multivariate linear regression analysis was conducted using the variables identified in univariate analysis. Collinearity statistics were computed to preclude concerns regarding multicollinearity between the predictor variables via the tolerance values and variance influence factor (VIF) statistics.

## Results

### Clinical and sociodemographic aspects of the study population

In total, 486 adult patients participated in this study. Appropriate, full datasets containing QOLIE-31 data were available for 476 patients (97.9% of all participants). The mean age of the cohort was 40.3 ± 15.4 years, and 58.6% (n = 279) were females. The most frequent epilepsy type was focal epilepsies (67.4%), 21.6% of patients had genetic generalized epilepsies, and the epilepsy type was unknown in 10.9% of patients. Patients took on average 1.8 ASMs (range 0–6). An overview of the relevant clinical and sociodemographic characteristics of the patients is provided in Table 1, and Table 2 presents the descriptive results of the assessed scores.

**Table 1:** Clinical and sociodemographic characteristics of the participants

Variable	Result
Age in years (n = 476), mean ± SD [range]	40.3 ± 15.4 [18–83]
Sex, n (%)	
Male Female	197(41.4) 279(58.6)
Epilepsy type, n (%)	
Focal Generalized Unknown	321(67.4) 103(21.6) 52(10.9)
Epilepsy onset in years (n = 457), mean ± SD [range]	24.1 ± 16.0 [0–79]
Mean duration of the epilepsy in years (n = 455), mean ± SD [range]	16.0 ± 15.1 [0–71]
Seizure frequency, n (%)	
daily weekly monthly once every 6 months once per year no seizure > 1 year n/a	21(4.4) 46(9.7) 85(17.9) 44(9.2) 51(10.7) 197(41.4) 32(6.7)
Number of anti-seizure medications (ASMs), n (%)	
0 1 2 ≥ 3 Number of ASMs, mean ± SD [range]	19(4.0) 195(41.0) 171(35.9) 91(19.1) 1.8 ± 0.9 [0–6]
Relationship status, n (%)	
Married/in a relationship Divorced/separated Single/living with relatives Living alone Widowed n/a	267(56.1) 23(4.8) 78(16.4) 93(19.5) 8(1.9) 7(1.5)
Has children, n (%)	
Yes No n/a	210(44.1) 260(54.6) 6(1.3)
Occupational situation, n (%)	
Working Stay-at-home Vocational training Unemployed Disability pension Retired Other or n/a	245(51.5) 22(4.6) 43(9.0) 30(6.3) 74(15.5) 36(7.6) 26(5.5)
Certificate of disability, n (%)	
No Yes n/a	199(41.8) 274(57.6) 3(0.6)
Patients taking antidepressant medication, n (%) (citalopram, escitalopram, fluoxetine, amitriptyline, duloxetine, sertraline, venlafaxine, mirtazapine)	32(6.7)
Patients currently receiving psychiatric treatment, n (%)	31(6.5)

**Table 2:** Descriptive statistics of the evaluated scores

Liverpool Adverse Events Profile (LAEP), (n = 471), mean ± SD [range]	38.3 ± 12.0 [19–72]
Neurological Disorder Depression Inventory for Epilepsy (NDDI-E), (n = 460), mean ± SD [range]	11.3 ± 4.3 [6–24]
- threshold for the presence of relevant depressive symptoms exceeded (score ≥ 14)	n = 129; 27.1%
Revised Epilepsy Stigma Scale (rESS), (n = 472), mean ± SD [range]	2.0 ± 2.5 [0–9]
Seizure Worry Scale (SWS), (n = 475), mean ± SD [range]	3.5 ± 1.8 [0–6]
Hospital Anxiety and Depression Scale (HADS) (n = 466), mean ± SD [range]	12.7 ± 6.5 [2–35]
HADS Anxiety	6.0 ± 4.2 [0–19]
HADS Depression	6.7 ± 2.9 [1–17]
- threshold for the presence of relevant depressive symptoms exceeded (HADS-D score ≥ 8)	n = 157; 33.0%
- threshold for the presence of relevant anxiety symptoms exceeded (HADS-A score ≥ 8)	n = 150; 26.8%

### EQ-5D – comparison with general population data

**Fig. 1:**
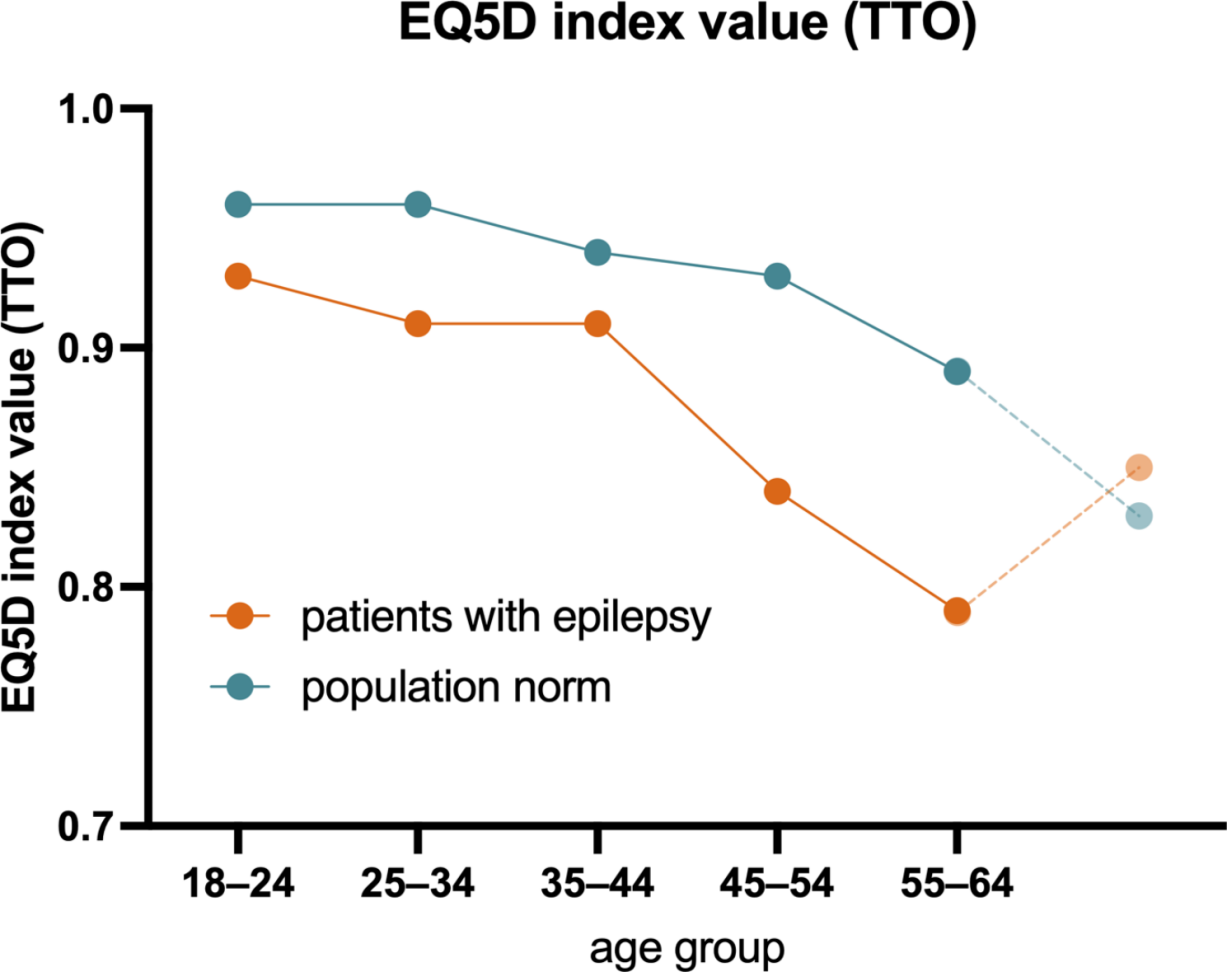
Representation of mean QOL values according to the EQ-5D index value time trade-off (TTO) in different age groups of PWE compared to normative data from the general population

PWE had lower EQ-5D scores than the population norm [15] in almost all age groups; the > 65-year-old group had the smallest number of participants (n = 37, 7.9%) (Fig. 1). In the epilepsy group, there was no significant difference in the EQ5D index values and overall QOLIE-31 scores between the individual age groups (p = 0.35).

### HADS – comparison with general population data

**Fig. 2:**
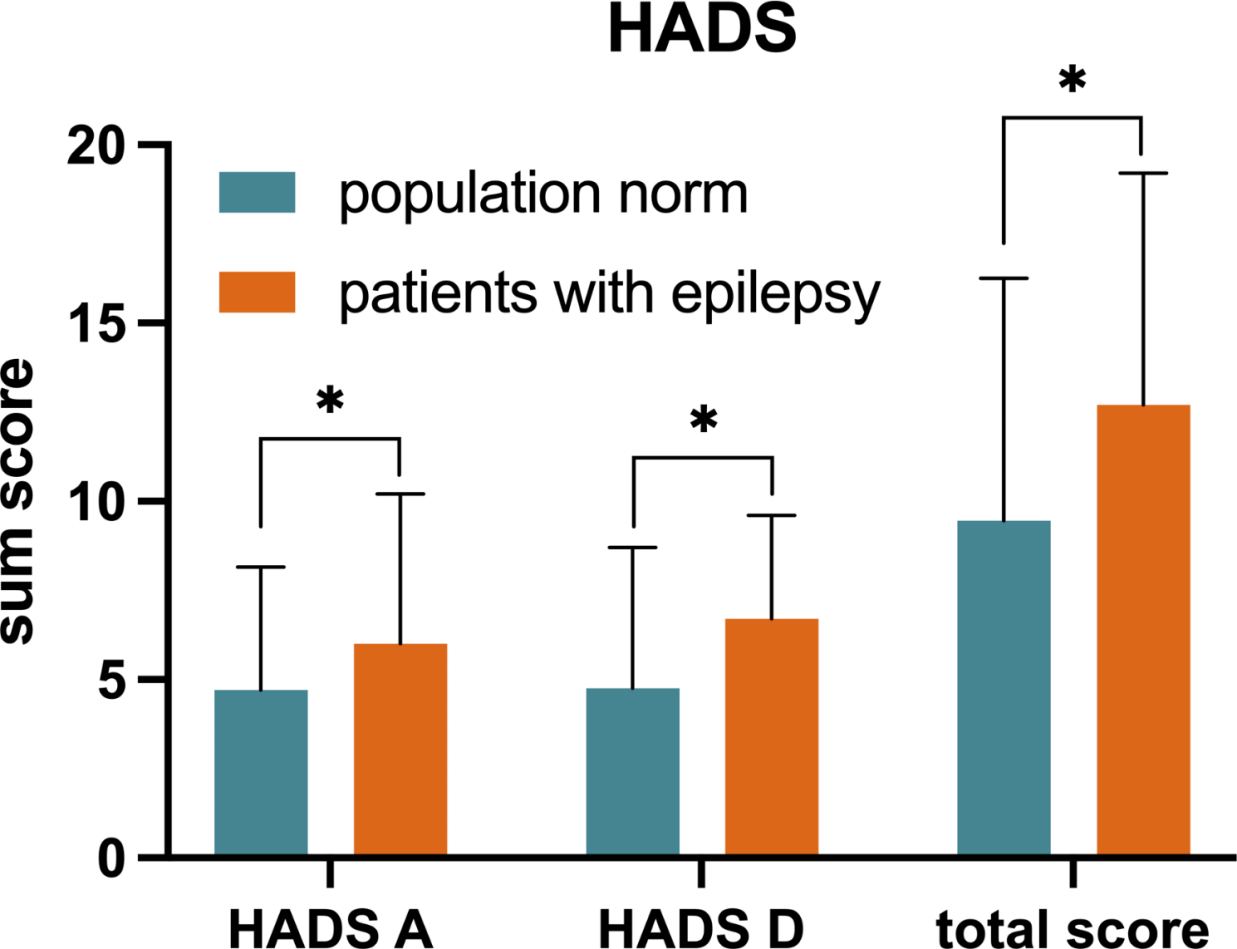
Representation of HADS scores in PWE and from normative data from the general population. Results are presented as mean values with SD. *P < 0.0001

PWE reported significantly higher HADS scores compared with the normative data from a German population (Fig. 2), with mean total scores of 12.7 ± 6.5 for PWE and 9.45 ± 6.8 for the representative sample of the German general population (p < 0.0001) [30].

### Associated factors with QoL in PWE in univariate analysis

**Fig. 3:**
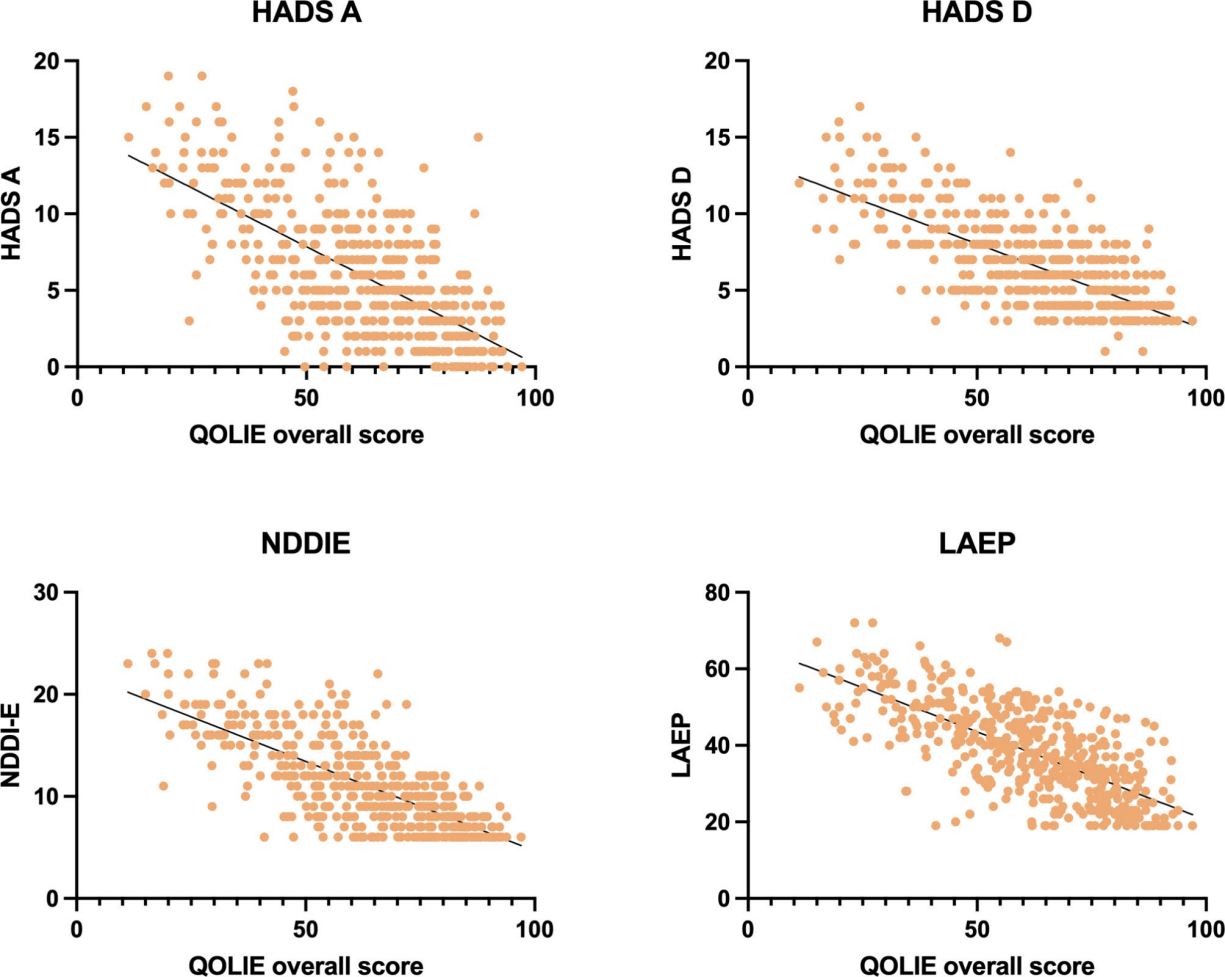
Univariate linear regression analysis of selected factors and their correlation with overall QOLIE score. All the correlations were highly significant (p < 0.001)

The mean overall QOLIE-31 score was 61.7, with a standard deviation of 18.4 (range: 11.2–97), and the mean QOLIE-31 visual analog scale value was 66.6 ± 18.3 (range: 0–100). Univariate analysis indicated that a low QOLIE-31 score was associated with a high BMI (p = 0.009), high number of ASMs (p < 0.001), more frequent and longer hospitalization (p < 0.001), high LAEP score (p < 0.001), rESS score (p < 0.001), SWS score (p < 0.001), NDDI-E score (p < 0.001), HADS-A score (p < 0.001), and HADS-D score (p < 0.001), employment status (p < 0.001), relationship status (p = 0.009), epilepsy type (p = 0.005), higher seizure frequency (p < 0.001), and the presence of a certificate of disability (p < 0.001). A graphical representation of selected factors is presented in Fig. 3.

Other examined variables, such as the age of the patients and their sex, having children and the duration of the epilepsy were not significantly correlated with QOLIE-31 scores.

### Association of seizure frequency on QoL and HADS

A higher seizure frequency was associated with lower QOLIE-31 total scores (p > 0.001), in comparison of seizure-free patients with those with persistent seizures, seizure-free patients showed significantly higher QOLIE-31 scores. When comparing the seizure-free patients with those with a low seizure frequency (up to one seizure every six months), there was no significant difference (p = 0.289).

Regarding the HADS scores, there was no significant difference between seizure-free patients and those with persistent seizures (p = 0.064).

### Multivariate linear regression analysis of predictor variables for QoL in PWE

The multivariate linear regression model reached an adjusted R^2^ of 0.78. Factors significantly associated with a low QOLIE-31 score were (in descending order of the standardized beta coefficient) a high LAEP score (beta=-0.28; p < 0.001), HADS-D score (beta=-0.27; p < 0.001), and NDDI-E score (beta=-0.19; p < 0.001). A high SWS score (beta = 0.18; p < 0.001), high seizure frequency (beta = 0.14; p < 0.001), and high rESS score (beta=-0.09; p = 0.027) were significantly correlated with overall QOLIE-31 score. The HADS-A score, hospitalization time, type of epilepsy syndrome, BMI, employment status, number of ASMs, relationship status, and the presence of a certificate of disability showed no significant association with QOLIE-31 scores. An overview of the multivariate linear regression model and the VIF is presented in Table 3.

**Table 3:** Results of multivariate regression analysis

Variable	Beta coefficient (stand.)	T	p-value	95% confidence interval	Beta	VIF
LAEP score	-0.282	-6.719	**< 0.0001**	− 0.562	-0.307	1.990
HADS-Depression score	-0.271	-6.329	**< 0.0001**	-2.280	-1.197	2.068
NDDI-E score	-0.193	-3.925	**0.0001**	-1.193	-0.396	2.715
Seizure Worry Scale	-0.180	-5.497	**< 0.0001**	-2.380	-1.124	1.215
Seizure frequency	0.139	3.950	**0.0001**	0.757	2.264	1.392
Revised Epilepsy Stigma Scale	-0.092	-2.229	**0.027**	-1.279	-0.079	1.929
HADS-Anxiety score	-0.066	-1.543	0.124	-0.654	0.079	2.074
Total hospitalization time in the last 3 months	-0.042	-1.280	0.202	-0.279	0.059	1.205
Epilepsy syndrome	0.036	1.117	0.265	-1.104	3.995	1.156
BMI	-0.027	− 0.861	0.390	-0.053	0.021	1.070
Employment status	0.023	0.647	0.518	-1.956	3.870	1.388
Number of ASMs	-0.020	− 0.577	0.564	-1.709	0.934	1.402
Relationship status	-0.019	− 0.607	0.545	-2.831	1.498	1.051
Presence of a certificate of disability	-0.008	− 0.242	0.809	-2.724	2.128	1.321

The values presented are the standardized beta-coefficients and their 95% confidence intervals, the T-value, corresponding p-value, and the variance influence factor (VIF). LAEP = Liverpool Adverse Events Profile, HADS = Hospital Anxiety and Depression Scale, NDDI-E = Neurological Disorders Depression Inventory for Epilepsy, BMI = body mass index, ASM = anti-seizure medication

## Discussion

This study was designed to identify risk factors of poor QoL in adult PWE using a cross-sectional, multicenter approach based on a large cohort of 476 adult patients. The present findings provide a broadened perspective of QoL in PWE and its associated factors, including disease-related, psychosocial, and psychobehavioral aspects.

The definition of QoL and the distinction between generic and health-related QoL have been discussed in the literature [31, 32]. Because the QOLIE-31 is a well-established tool used to measure epilepsy-related limitations, the results of this study regarding this parameter represent health-related QoL. The results that represent values of the EQ-5D as a generic tool are regarded as a parameter of QoL.

These findings suggest that PWE have a lower generic QoL compared with the normative population data, as evidenced by the EQ-5D scores. In addition, PWE have a higher likelihood of having concomitant psychiatric comorbidities, especially depression, which confirms the findings of previous studies [33, 34]. Health-related QOL in PWE, as represented by QOLIE-31 scores, is not age-dependent [35]. Several factors were identified as being significantly associated with poor QoL using a multivariate linear regression model with a high adjusted R^2^ of 0.78.

### Disease-related factors

Following multivariate linear regression analysis, the factor with the highest impact in predicting lower QOLIE-31 scores was a high LAEP score, indicating the influence of medication-related side effects on QoL. This is in line with the result of several studies that reported a negative influence of adverse events due to ASMs on QoL, especially in combination with psychiatric comorbidities [36]. The results of the LAEP, HADS-D, and NDDI-E scores overlapped, a recent mediation analysis revealed that patient-reported depression and ASM-related adverse events jointly mediated the association between ASMs and QoL [37]. Adverse events must be carefully evaluated, and the adverse events profile should always be taken into consideration when selecting an ASM [38]. The number of ASMs was not significantly correlated with QoL. There is a lack of consensus in the literature about the roles of monotherapy and polytherapy [39]. Our data suggest that poor QoL related to pharmacotherapy is due to the side effects of the medications, regardless of the number of ASMs, and patients receiving polytherapy have a higher risk of adverse events. Therefore, polytherapy with moderate doses and a moderate risk of side effects could improve QoL compared with fewer ASMs with high doses and a higher risk of side effects.

In addition, this study confirms that poor seizure control with high seizure frequency is significantly associated with low QoL. Seizure-freedom is associated with higher QoL, although there is no significant difference in seizure-free patients compared to those with a low seizure frequency (up to one seizure every six months). The likely causes of poorer QoL related to a higher seizure frequency are restrictions in everyday life, like driving a car [40], seizure-related injuries[8], and the fear of new seizures [41], highlighting the need for the adequate use of ASMs or epilepsy surgery, as QoL has been reported to improve following epilepsy surgery [42]. The effect of seizure frequency on QoL could also be explained by the possibility of reducing ASM numbers and doses to decrease the severity and number of side effects.

The type of epilepsy syndrome, duration of epilepsy, and hospitalization time as direct disease-related features were not significantly correlated with QOLIE-31 score. This observation highlights that the prevalence of epilepsy as a chronic disease is not inevitably associated with low QoL.

### Psychiatric comorbidities

Depressive mood disorders and other neuropsychiatric comorbidities are common and represent a well-known risk factor for reduced QoL [43]. In this study, PWE had significantly higher HADS scores compared with the normative population data. Furthermore, a highly significant correlation was observed between NDDI-E and HADS-D scores and a poor overall QOLIE-31 score, confirming the influence of mood disturbances on individual QoL. The seizure frequency showed no significant correlation to the HADS scores, suggesting that also seizure-free patients have an increased risk of psychiatric comorbidities. Regarding the standardized beta coefficients in the multivariate analysis, the depression scales NDDI-E and HADS-D showed a higher association with QoL than the seizure frequency, confirming previous studies that showed that depression was a much stronger predictor of QoL than seizure frequency [44]. Although they are supposed to measure the same parameter, the NDDI-E and HADS-D did not exhibit collinearity in our study. In a previous comparison of the tests, the NDDI-E and HADS-D were considered brief but efficient screening instruments to identify depression in PWE. For identifying suicide risk, the sensitivity of the NDDI-E was slightly higher than that of the HADS-D [45].

Considering the individual NDDI-E and HADS-D results, 27.1% (NDDI-E) and 33.0% (HADS-D) of the study population exceeded the threshold for the presence of relevant depressive symptoms; however, only 6.7% were taking antidepressant medications and only 6.5% had received psychiatric or psychotherapeutic treatment. This implies that the diagnosis of comorbid depression is insufficient, which is in line with other studies on QoL and depression [46], and the lack of adequate therapy may contribute to a lower QoL. The Psychology Task Force of the International League Against Epilepsy concluded that psychological therapies that target comorbid mental health symptoms, especially depression, should be considered in the comprehensive treatment of PWE. Treatments for these disorders and conditions have received strong recommendations [47].

Anxiety has been described as a factor that influences poor QoL, albeit much less frequently than depression [48]. Our data did not indicate a significant correlation between poor QoL and anxiety represented by HADS-A and QOLIE-31 scores.

### Psychosocial factors

Another factor that is associated with low QoL is individual fear about the occurrence of new seizures, which was represented by the SWS. A high SWS score was significantly correlated with a low QOLIE-31 score. Supporting therapy involving psychological interventions can improve QoL [49]. Furthermore, the individual perception of stigmatization plays a key role in QoL, and the results of this study indicate a significant correlation between the perception of stigma and QoL. The literature describes the stigmatization experienced by PWE [50], their relatives and caregivers [51]. Therefore, the identification and sufficient management of psychiatric comorbidities in addition to individual worry and stigmatization can help to decrease comorbid psychopathology in PWE and thereby enhance individual QoL.

Other clinical, psychosocial, and socioeconomic factors, like sex, BMI, employment status, relationship status, having children, and the presence of a certificate of disability, were not significantly correlated with QoL following the multivariate analysis.

### Limitations

The use of a cross-sectional study design allowed the researchers to highlight significant correlations; however, no causal relationships could be drawn between the analyzed disease-related aspects and individual QoL. The study population was recruited from specialized epilepsy centers; therefore, the population had a higher degree of pharmacoresistance than the general epilepsy population, which may have introduced selection bias; however, this may have been reduced through the use of a multi-center approach. The study design included all patients with a diagnosis of epilepsy, leading to a heterogeneous population. Another limitation is that the self-reported QoL and health-related scores represented the patients’ subjective perceptions, especially for psychiatric comorbidities, as no specific psychiatric consultations were performed. Finally, the data acquisition occurred during the COVID-19 pandemic, and the pandemic-related restrictions in Germany may have represented an additional source of bias [52].

## Conclusion

PWE have reduced QoL and are more likely to suffer from mood disorders than the general population in Germany. A variety of factors are associated with decreased QoL in PWE. In addition to disease severity, as measured by seizure frequency, the patient’s tolerability of ASMs and the presence of depression, seizure worry, and stigmatization were strongly associated with poor individual QoL. Depression was underrepresented, indicating a need for better diagnosis and therapy.

The goal of epilepsy treatment is to achieve seizure freedom. In chronic diseases, it is also important to consider the patient’s concomitant circumstances to provide them with the highest possible QoL. Therefore, therapeutic decisions should always be made with the patient’s input and in consideration of individual psychobehavioral and disease-specific aspects. Screening tools, like the NDDI-E and HADS, are easily accessible and can aid in the diagnosis of depression. Signs of ASM-related adverse events, depressive symptoms, and stigmatization should be actively sought to ensure that PWE receive personalized and optimized treatment to improve their QoL.

## Data Availability

The datasets generated and/or analyzed during the current study are not publicly available due to national data protection laws but are available from the corresponding author upon reasonable request.

## List of abbreviations

ASM: anti-seizure medication
BMI: body mass index
EQ5D: European Quality of Life 5 Dimensions
HADS: Hospital Anxiety and Depression Scale
HADS-A: Hospital Anxiety and Depression Scale–Anxiety subscale
HADS-D: Hospital Anxiety and Depression Scale–Depression subscale
ILAE: International League Against Epilepsy
LAEP: Liverpool Adverse Events Profile
NDDI-E: Neurological Disorders Depression Inventory for Epilepsy
PWE: patients with epilepsy
QOLIE-31: 31-item Quality of Life in Epilepsy inventory
rESS: revised Epilepsy Stigma Scale
SWS: Seizure Worry Scale
